# 

**Published:** 1981

**Authors:** 


### BRISTOL MEDICO-CHIRURGICAL JOURNAL

**JANUARY/APRIL 1981**
**VOLUME 96 (i/ii)**
**NUMBER 357/358**

